# Prevalence and antibiotic susceptibility pattern of methicillin-resistant *Staphylococcus aureus* (MRSA) among primary school children and prisoners in Jimma Town, Southwest Ethiopia

**DOI:** 10.1186/1476-0711-12-11

**Published:** 2013-06-04

**Authors:** Tekalign Kejela, Ketema Bacha

**Affiliations:** 1 Department of Biology, College of Natural Sciences, Jimma University, Jimma, Ethiopia; 2 Department of Biology, College of Natural and Computational Sciences, Mettu University, Mettu, Ethiopia

**Keywords:** CA-MRSA, Drug resistance, Ethiopia, HA-MRSA, Prevalence, Risk factor, *S. aureus*

## Abstract

**Background:**

*Staphylococcus aureus* infections are increasingly reported from both health institutions and communities around the world. In particular, infections due to methicillin-resistant *Staphylococcus aureus* (MRSA) strains have been detected worldwide. If MRSA becomes the most common form of *S. aureus* in a community, it makes the treatment of common infections much more difficult. But, report on the current status of community acquired MRSA in the study area is scanty.

**Methods:**

Community-based cross sectional study was conducted to evaluate the current prevalence and antibiotic susceptibility pattern of MRSA among primary school children and prisoners in Jimma town. MRSA was detected using Cefoxitin (30μg) disc; and epidemiologic risk factors were assessed using pre-designed questionnaires distributed to the children’s parents and prisoners. A total of 354 nasal swabs were collected from primary school children and prisoners from December 2010 to March 2011 following standards microbiological methods.

**Results:**

A total of 169 *S. aureus* isolates were recovered. The overall prevalence of MRSA among the study population was 23.08 % (39/169). Specifically, the prevalence of MRSA among primary school children and prisoners were 18.8% (27/144) and 48% (12/25), respectively. The isolated *S. aureus* and MRSA displayed multiple drug resistance (MDR) to 2 to 10 antibiotics. The most frequent MDR was Amp/Bac/Ery/Pen/Fox (resistance to Ampicillin, Bacitracin, Erythromycin, Penicillin, and Cefoxitin).

**Conclusion:**

The present study revealed that MRSA could be prevalent in the healthy community, transmitted from hospital to the community. The high distribution of MRSA could be favored by potential risk factors. Thus, for comprehensive evaluation of the current prevalence of MRSA and design control measures, consideration need to be given to the healthy community besides data coming from health institutions.

## Introduction

*Staphylococcus aureus* is a Gram positive bacteria commonly found on the skin and in the nose of 30% of healthy people [1,2]. It is a leading cause of human bacterial infections worldwide [1]. This infection can be minor (such as pimples, boils, and other skin conditions) or serious and sometimes fatal (such as blood infections or pneumonia). Person-to-person transmission is the usual form of spread and occurs through contact with secretions from infected skin lesions, nasal discharge or spread via hands.

*S. aureus* has outstanding ability to acquire resistance to antibiotics. Benzyl penicillin was no longer effective for treatment of most *S. aureus* infections within 10 years after its introduction for use because of the acquisition of plasmid-encoded β-lactamase. Penicillin-resistant *S. aureus* became pandemic throughout the late 1950s and early 1960s. Methicillin is β-lactam antibiotic invented to treat Penicillin resistant *S. aureus*. However, Meticillin-resistant *S. aureus* (MRSA) was reported 2 years after the antibiotic was introduced in 1961 in the United Kingdom [3].

The increasing frequency of antimicrobial resistance among infectious organisms is of great concern to both medical providers and the general public [4]. Methicillin-resistant *S. aureus* (MRSA) is any strain of *S. aureus* bacterium that is resistant to a large group of antibiotics called the beta-lactams. As MRSA has adapted to survive treatment with antibiotics, including Methicillin, Dicloxacillin, Nafcillin, and Oxacillin, it may also be referred to as multidrug-resistant *S. aureus* or Oxacillin-resistant *S. aureus* (ORSA). MRSA is a bacterium responsible for several difficult-to-treat infections in human. It is an emerging type of *S. aureus*[3] implicated in skin and soft tissue infections [4]. MRSA also causes abscess in deep organs, and responsible for toxin mediated diseases.

Now days, MRSA is the hottest research area in most developed countries due to increased mortality and morbidity. The 2007 report on Emerging Infectious Diseases estimated that the number of MRSA infections in hospitals has doubled nationwide, from approximately 127,000 in 1999 to 278,000 in 2005, while at the same time annual deaths increased from 11,000 to more than 17,000 [5]. Another report estimated that MRSA has been responsible for 94,360 serious infections and associated with 18,650 hospital stay-related deaths in the United States in 2005 [6]. Accordingly, MRSA infections are responsible for more deaths in the US each year than AIDS. The Office for National Statistics of England reported 1,629 MRSA-related deaths in England and Wales during 2005 [2]. MRSA is thought to have caused 1,652 deaths in 2006 in UK which was much higher than the 51 cases in 1993 [7]. Similarly, higher isolation rate of MRSA has been reported from most African countries including Nigeria (29.6%), Kenya (27.7%), Cameroon (21.3%), Cote D’Ivoire (16.8%), and Morocco (14.4%) [8].

In Ethiopia, 42.8% isolation rate of MRSA was reported from among health workers of Jimma Specialized Hospital [9]. This raises serious concerns about the possibility of transmission of MRSA outside the health care system. If MRSA becomes the most common form of *S. aureus* in a community, it will make treatment of common infections much more difficult [4]. In general, most of the data related to the prevalence of MRSA in Ethiopia are restricted to health institution, hence Hospital Acquired MRSA (HA-MRSA) for which there are an already known predisposing risk factor or illness. However, there are great chances of MRSA to be transmitted outside hospital for the fact that there is no checkup for MRSA decolonization when patients are discharged from hospitals. This necessitates the need for evaluation of whether the pathogen is restricted in hospitals or already disseminated to the healthy community members. Therefore, this study was initiated to determine the prevalence and antibiotic susceptibility pattern of MRSA among healthy Primary School children and prisoner in Jimma town.

## Materials and methods

### Study area

The study was conducted in Jimma town, southwest Ethiopia. Geographically, the town is located at an altitude ranging from 1700–1750 meter above sea level, 07^o^40’ Latitude and 36^o^50’ longitude. According to the 2005 national census, the total population of the town was 151,679. In the town, there are 27 primary schools of which 13 are governmental and 14 are non-governmental (private) schools. There was only one prison center in the town. The study was conducted from December 2010 to June, 2011.

### Sample size and sampling technique

A total of 354 participants were recruited for this study: 304 primary school children and 50 prisoners. Multistage sampling technique was employed to randomly select primary school children at school type, grade and section level. Prisoners were selected by systematic random sampling method. Accordingly, the attendance list of 1281 prisoners was checked and the first person was selected by lottery method from the first 26 (1281/50) individuals in the list. Selections of the other 49 prisoners were made every 26 individual based on the roll number of the 1^st^ selected prisoner. The sample size was shared among the primary school children and the prisoners based on the total population of the two study populations.

### Data collection for epidemiologic risk factors

Epidemiologic risk factors related to demographic and medical history of the study population (antibiotic usage in the past 4 weeks, the completion of taking the prescribed antibiotic, presence of respiratory infection, previous year hospitalization of self or family member, having chronic disease and having member of medical staff in his/her family) were gathered using pre-designed structured questionnaires a day before sample collection.

### Microbiological study

Collection, handling and transporting of samples of nasal swabs were carried out according to Rijal et al. [10]. Briefly, nasal swabs from the study participants were collected using sterile cotton swabs, by rotating 4–5 times both clockwise and anticlockwise before withdrawal. The swabs were inoculated into transport medium, labeled and transported to laboratory within an hour of its collection using cold chain. Standard reference strains including *Staphylococcus aureus* (ATCC 25923)*, E. coli (*ATCC 25922) and *Streptococcus spp*. were kindly obtained from Ethiopian Health and Nutrition Research Institute (EHNRI), Addis Ababa, and used as a quality control organisms.

Isolation and identification of *S. aureus* was carried out following a standard microbiological method as used by Cheesbrough [11]. A control strain of *S. aureus* (ATCC 25923) was also inoculated separately into culture media and used with every batch of samples. Characteristic colonies of *S .aureus* (round, convex, golden yellow, mannitol fermenting colonies on MSA plates) were aseptically picked, further purified by repeated streaking on Nutrient agar plate and characterized following established microbiological methods (evaluation for colonial morphology, cell shape and grouping, Gram reaction, catalase and coagulase tests).

### Test for antimicrobial susceptibility patterns

The antimicrobial susceptibility patterns of the isolates were determined according to Kirby Bauer disc diffusion technique as described by Clinical Laboratory Standard Institute (CLSI) [11]. The following 12 antibiotics and concentrations were used to determine the antibiogram of the isolates: Ampicillin (10μg), Amikacin (10 μg), Bacitracin (10unit), Chloramphenicol (30 μg), Erythromycin (15 μg), Gentamicin (10 μg), Kanamycin (30 μg), Penicillin G (10 unit), Trimethoprim-sulfamethoxazole (25μg), Tetracycline (30 μg) and Vancomycin (30 μg). Standardized overnight culture suspension (0.5 McFarland or barium sulphate solution equivalents) of the test organism was used to swab the surface of sterile Mueller-Hinton agar plates. A set of 6 standard antimicrobial discs were then placed aseptically on the inoculated Mueller Hinton agar plates and allowed to stand for 30 minutes. The inoculated plates were incubated at 35^0^C for 16-18h. The diameter of the zone of inhibition (mm) of each antimicrobial disc was measured, and the isolates were classified as resistant, intermediate or susceptible following the standard interpretive chart of CLSI / NCCLS [11].

### Detection of methicillin resistance

Detection of Methicillin resistant *S. aureus* (MRSA) was carried out using Cefoxitin disc as described earlier [12]. Briefly, all the isolates were subjected to Cefoxitin disc diffusion test using a 30μg disc. A 0.5 McFarland standard suspension of the isolates were made and a lawn culture was done on Mueller Hinton agar plate. Plates were incubated at 35^0^C for 18–24 hours and inhibition zone diameters (mm) were measured. An inhibition zone diameter of ≤ 21mm was reported as methicillin/oxacillin resistant and ≥ 22mm was considered as methicillin /oxacillin sensitive[12].

### Data quality management

At regular intervals and whenever a new batch of stain or reagent is prepared, standard strain of *S. aureus* ATCC 25923 was used as positive control for catalase, coagulase and antibiotic susceptibility test, to ensure data quality. *Streptococcus* species and *E. coli* were used as negative control of the catalase test and coagulase test, respectively. The sterility of used media was checked by incubating the media overnight before its use.

### Statistical analysis

SPSS for windows (Version 13.0; SPSS, Chicago, III, USA) software was used for the statistical analysis of the collected data. Frequency and percentage were presented for categorical data. Chi-square test was applied to determine potential risk factors associated with *Staphylococcus* nasal carriage. The level of significance was set at α = 0.05 using the two-tailed method.

### Ethical considerations

Ethical permission was obtained from the Research Review and Ethical Committee of College of Natural Sciences, Jimma University. The objectives as well as the nature of the study were explained to the school community, parents, and prisoners for purpose of their consent.

## Result

### Age and sex distribution of the study participants

About 51% (155/304) of the school children involved in the study were aged between 11–15 years, and 51.3% (156/304) were females (Table 1). Likewise, 58% (29/50) of the prisoners were younger than 25 years while 20% (10/50) were older than 36 years.

**Table 1:** Age and sex distribution of school children participated in the study in each school and school type, Jimma town, 2011

** Schools**		**Age of children (Year) (N=304)**				**Sex of children (N=304)**			
		**5-10**		**11-15**		**Female**		**Male**	
		**No.**	**%**	**No.**	**%**	**No.**	**%**	**No.**	**%**
School Name	Ginjo^1^	53	44.9	65	55.1	62	52.5	56	47.5
	Hermata^1^	59	50.4	58	49.6	61	52.1	56	47.9
	JU Community sch^2^	13	54.2	11	45.8	12	50.0	12	50.0
	Eldan^2^	24	53.3	21	46.7	21	46.7	24	53.3
	Total	149	49.0	155	51.0	156	51.3	148	48.7
School Type	Government	112	47.7	123	52.3	123	52.3	112	47.7
	Non-government	37	53.6	32	46.4	33	47.8	36	52.2
Total		149	49.0	155	51.0	156	51.3	148	48.7

*N* total number of children participated in the study; ^1^public/governmental schools; ^2^Private/Non-governmental schools, *Sch* School.

### Frequency of isolation of ***S. aureus***

From a total of 354 nasal swabs collected from 304 primary school children and 50 prisoners, the overall frequency of isolation of *S. aureus* was 47.74% (169/354) and that of coagulase-negative *Staphylococcus* (CNS) was 52.26% (185/354) (Table 2). With respect to sources of sample, the isolation rate of *S. aureus* among primary school children and prisoners were 47.34% (144/354) and 50% (25/50), respectively (Table 2). Among the primary school children, high rate of isolation of *S. aureus* was observed in Hermata Primary School (50.4%, 59) followed by Ginjo (46.6%, 55), Eldan (44.4%, 20), and Jimma University Community Primary School (41.7%, 10). As compared to the primary school children, the isolation rate of *S. aureus* was the highest (50%, 25) among prisoners (Table 2). Rate of isolation of *S. aureus* from government and non-government primary schools were 114(48.5%) and 30(43.5%), respectively, with only 5% higher rate of isolation among government primary schools (Table 2). Thus, the rate of isolation of *S. aureus* was not significantly different between types of schools (p=0.495) as well as schools and detention center.

**Table 2:** **Frequency of isolation of *S. aureus *from primary school children and prisoners, Jimma town, 2011**

** Source of the samples**	**No. of positive samples**	***Staphylococcus *isolates**				**p-value**
		***S. aureus***		**CNS**		
		**No.**	**(%)**	**No.**	**(%)**	
Eldan^2^	45	20	44.4	25	55.6	0.495
JU Community school ^2^	24	10	41.7	14	58.3	
Sub-Total	69	30	43.5	39	56.5	
Ginjo^1^	118	55	46.6	63	53.4	
Hermata^1^	117	59	50.4	58	49.6	
Sub-total	235	114	48.5	121	51.5	
Total of positive	304	144	47.37	160	52.63	
School samples						
Jimma zone detention center	50	25	50	25	50	
Grand total	354	169	47.74	185	52.26	

^1^public/governmental schools; ^2^Private/Non-governmental schools; *CNS* Coagulase-negative *Staphylococcus.*

### Prevalence of methicillin-resistant ***S. aureus***

Of the total 169 *S. aureus* isolate, 39(23.08%) were found resistant to methicillin/ Cefoxitin (Table 3). Maximum nasal carriages of MRSA were observed among the prisoners (48%, 12). Overall prevalence of MRSA among primary school children was 18.8% (n=27). Of the four primary schools children, rate of isolation of MRSA was the highest among Hermata primary school children (20.3%, 12), followed by Eldan (20%, 4), Ginjo (18.2%, 10) and JU Community primary school children (10%, 1). Among the school type, the isolation rate was the highest in government primary schools 22(19.3%) than that of non-governmental primary schools 5(16.7%) (Table 3). However, there was no statistically significant difference in isolation rate of MRSA nasal carriage between school types (p=0.742)

**Table 3:** Prevalence of MRSA among primary school children and prisoners, Jimma town, 2011

** Sampling site**	***S. aureus*isolate**				***P-value***
	MRSA* (N=39)		MSSA** (n=130)		
	**No**	**(%)**	**No.**	**(%)**	
Eldan^2^	4	20	16	80	0.742
JU community school^2^	1	10	9	90	
Sub-total	5	16.7	25	83.3	
Ginjo^1^	10	18.2	45	81.8	
Hermata^1^	12	20.3	47	79.7	
Sub-total	22	19.3	92	80.7	
Total of positive	27	18.8	117	81.2	
School samples					
Jimma Zone detention center	12	48	13	52	
Grand total	39	23.08	130	76.92	

^1^public/governmental schools; ^2^Private/Non-governmental schools; *MRSA, Methicillin Resistant *S. aureus*; **MSSA Methicillin Sensitive *S. aureus, JU* Jimma University.

### Evaluation of antibiotic susceptibility test

A total of 169 *S. aureus* isolates were subjected to antibiotic susceptibility test against 12 antimicrobial drugs. Majority of the *S. aurous* isolates were sensitive to Vancomycin (97%, 164) followed by Gentamycin (95.3%, 161), Trimethoprim-sulfamethoxazole (94.7%, 160), Amikacin (79.3%, 134), Tetracycline (79.3%, 134), Cefoxitin (76.9%, 130), Erythromycin (72.2%, 122), and Kanamycin (65.1%, 110) (Table 4). However, no isolate was sensitive to Penicillin. Accordingly, the highest resistance were observed for Penicillin (100%, 169) followed by Ampicillin (76.3%, 129), and Chloramphenicole (65.7%, 62) (Table 4). The least resistance was observed for Vancomycin with only 3% resistance recorded. About quarter (23.1%, 39) of the *S aureus* isolates were resistant to Cefoxitin, which represent MRSA. On the other hand, all the 39(100%) isolates of MRSA were resistant to Ampicillin, Cefoxitin and Penicillin G (Table 4). Similarly, the highest susceptibility of MRSA isolates were observed for Vancomycin (87.2%, 34) followed by Genatmycin (84.6%, 33), Trimethoprim-sulfamethoxazole (82.1%, 32), Amikacin (71.8%, 28) and Tetracycline (66.7%, 26) (Table 4). Except for Chloramphenicole (p=0.528) and Tetracycline (p=0.076), the susceptibility of all the other antibiotics have statistically significant association with MRSA (p<0.05) (Table 4). Of the total of 130 MSSA isolates, the highest resistance were observed for Penicillin (100%, 130) followed by Ampicillin (69.2%, 90) and Chloramphenicole (67.7%, 88) (Table 4).

**Table 4:** **Antibiotic susceptibility patterns of *S. aureus *and MRSA isolates, Jimma town, 2011**

**Antimicrobial drugs**		***S. aureus***				**Total**		**P-value**
		MRSA* N=39		MSSA** n=130				
		**No.**	**%**	**No.**	**%**	**No.**	**%**	
Ampicillin	*Susceptible*	*-*	*-*	*40*	*30.8*	*40*	*23.7*	*0.000*
	*Resistant*	*39*	*100*	*90*	*69.2*	*129*	*76.3*	
Amikacin	*Susceptible*	*28*	*71.8*	*106*	*81.5*	*134*	*79.3*	*0.017*
	*Resistant*	*11*	*28.2*	*24*	*18.5*	*35*	*20.7*	
Bacitracin	*Susceptible*	*15*	*38.5*	*92*	*70.8*	*107*	*63.3*	*0.000*
	*Resistant*	*24*	*61.5*	*38*	*29.2*	*62*	*36.7*	
Chloramphenicol	*Susceptible*	*16*	*41.0*	*42*	*32.3*	*58*	*34.3*	*0.528*
	*Resistant*	*23*	*59*	*88*	*67.7*	*111*	*65.7*	
Erythromycin	*Susceptible*	*15*	*38.5*	*107*	*82.3*	*122*	*72.2*	*0.000*
	*Resistant*	*24*	*61.5*	*23*	*17.7*	*47*	*27.8*	
Gentamicin	*Susceptible*	*33*	*84.6*	*128*	*98.5*	*161*	*95.3*	*0.006*
	*Resistant*	*6*	*15.4*	*2*	*1.5*	*8*	*4.7*	
Kanamycin	*Susceptible*	*14*	*35.9*	*96*	*73.8*	*110*	*65.1*	*0.000*
	*Resistant*	*25*	*64.1*	*34*	*26.2*	*59*	*34.9*	
Penicillin G	*Resistant*	*39*	*100*	*130*	*100*	*169*	*100*	*-*
Trimethoprim-sulfamethoxazole	*Susceptible*	*32*	*82.1*	*128*	*98.5*	*160*	*94.7*	*0.000*
	*Resistant*	*7*	*17.9*	*2*	*1.5*	*9*	*5.3*	
Tetracycline	*Susceptible*	*26*	*66.7*	*108*	*83.1*	*134*	*79.3*	*0.076*
	*Resistant*	*13*	*33.3*	*22*	*16.9*	*35*	*20.7*	
Vancomycin	*Susceptible*	*34*	*87.2*	*130*	*100.0*	*164*	*97.0*	*0.000*
	*Resistant*	*5*	*12.8*	*-*	*-*	*5*	*3*	
Cifoxitin	*Susceptible*	*-*	*-*	*130*	*100.0*	*130*	*76.9*	*0.000*
	*Resistant*	*39*	*100*	*-*	*-*	*39*	*23.1*	

*N* total number of MRSA isolates, *n* total number of MSSA isolates.* Methicillin Resistant *S. aureus;* ** Methicillin Susceptible *S. aureus.*

### Multi-drug resistance in ***S. aureus*** isolate

The *S. aureus* isolates showed multiple drug resistance (MDR) patterns. The highest MDR pattern (2.37%) was observed for five antibiotics with the pattern Ampicillin /Bacitracin / Erythromycin / Penicillin / Cefoxitin (Table 5). Five resistance patterns of equal proportion were observed for resistance to six antibiotics including the pattern such as Ampicillin /Bacitracin/Erythromycin / Penicillin / Vancomycin / Cefoxitin. Only two resistance patterns (Ampicillin/Amikacin / Chloramphenicol / Erythromycin /Kanamycin / Penicillin / Cefoxitin and Ampicillin /Bacitracin /Chloramphenicol /Erythromycin /Penicillin /Trimethoprim-sulfamethoxazole/Cefoxitin) were the case for resistance to seven antibiotics. The maximum number of antibiotics resisted by single isolate was ten antibiotics (Table 5) with MDR pattern of Amp / Amk / Bac /Chl /Ery/Kan /Pen /SXT/Tet /Fox (resistance to ampicillin, amikacin, bacitracin, chloramphenicol, erythromycin, kanamycin, penicillin, Trimethoprim-sulfamethoxazole, tetracycline and Cefoxitin).

**Table 5:** **Multiple drug resistance patterns of *S. aureu*s isolates that resisted 5 to 10 antibiotics, Jimma town, 2011**

**Number of antibiotics towards which resistance developed**	**MDR pattern**	**No and % resistant strains**	
		**No**	**%**
5	Amp/Amk/Chl/Kan/Pen	1	0.59
	Amp/Amk/Gen/Kan/Pen	1	0.59
	Amp/Bac/Ery/Pen/Fox	4	2.37
	Amp/Bac/Kan/Van/Fox	1	0.59
	Amp/Kan/Pen/Tet/Fox	1	0.59
	Amp/Pen/Tet/Van/Fox	1	0.59
6	Amp/Bac/Ery/Pen/Van/Fox	1	0.59
	Amp/Amk/Chl/Ery/Pen/Fox	1	0.59
	Amp/Amk/Bac/Ery/Pen/Fox	1	0.59
	Amp/Bac/Ery/Gen/Pen/ Fox	1	0.59
	Amp/Bac/Chl/Ery /Pen/ Fox	1	0.59
7	Amp/Amk/Chl/Ery/Kan/Pen/Fox	1	0.59
	Amp/Bac/Chl/Ery/Pen/SXT/Fox	1	0.59
8	Amp/Amk/Ery/Kan/Pen/SXT/Tet/Fox	1	0.59
	Amp/Amk/Chl/Ery/Gen/Kan/Pen/ Fox	1	0.59
9	Amp/Amk/Bac/Chl/Ery/Kan/Pen/SXT/Fox	1	0.59
	Amp /Bac/Chl/ Ery/Gen/Pen/SXT/Tet/ Fox	1	0.59
10	Amp / Amk / Bac /Chl /Ery/Kan /Pen /SXT/Tet /Fox	1	0.59

Where: *Amp* Ampicillin (10μg), *Amk* Amikacin (10μg), *Bac* Bacitracin (10unit), *Chl* Chloramphenicol (30μg), *Fox* Cefoxitin, *Ery* Erythromycin (15μg), *Gen* Gentamicin (10μg), *Kan* Kanamycin (30 μg), *Pen* Penicillin G (10 unit), *SXT* Trimethoprim-sulfamethoxazole (25μg), *Tet* Tetracycline (30μg) and *Van* Vancomycin (30μg), *MDR* multiple drug resistant.

### Epidemiologic risk factors for MRSA

Nasal carriage of *S. aureus* among female and male children were 73(50.7%) and 71(49.3%), respectively. The isolation rate of MRSA was higher among females 23(85.2%) as compared to that of males 4(14.8%). Nasal carriage of *S. aureus* was insignificantly associated to sex of the children (p=0.909) while MRSA nasal carriage was significantly association to sex of the children (p= 0.000) (Table 6). Among 144 children with *S. aureus,* 18(12.5%) of the children’s family member had history of previous year hospitalization (Table 6) while the. majority (87.5%, 120) had no family member who were hospitalization in the past 1 yrs. Similarly, of the 27 nasal carriers of MRSA, (29.6%, 8) of the children family members had history of previous year hospitalization although the majority’s (70.4%, 19) family members had no any history of previous year hospitalization. Nasal carrier of MRSA among children were significantly associated to family members’ previous year hospitalization (p=0.007) while *S. aureus* nasal carriage had no significant association to previous year’s hospitalization (p=0.289).

**Table 6:** **Analysis of epidemiologic risk factors for *S. aureus *and MRSA nasal carriage among primary school children, Jimma town, 2011**

**Risk factors**		***Staphylococcus***				**P-value**	**Methicillin resistance *S.aureus***				**P-value**
		***S.aureus***		**CONS**			**Positive**		**Negative**		
		**NO.**	**%**	**NO.**	**%**		**NO.**	**%**	**NO.**	**%**	
Age	5-10	*86*	*59.7*	*63*	*39.4*	*0.001*	*21*	*77.8*	*65*	*55.6*	*0.049*
	11-15	*58*	*40.3*	*97*	*60.6*		*6*	*22.2*	*52*	*44.4*	
Sex	Female	*73*	*50.7*	*83*	*51.9*	*0.909*	*23*	*85.2*	*50*	*42.7*	*0.000*
	Male	*71*	*49.3*	*77*	*48.1*		*4*	*14.8*	*67*	*57.3*	
Hospitalization in the past 1 years	Present	*9*	*6.3*	*8*	*5.0*	0.803	*4*	*14.8*	*5*	*4.3*	0.041
	Absent	*135*	*93.8*	*152*	*95.0*		*23*	*85.2*	*112*	*95.7*	
Antibiotic usage in the past 4 weeks	Present	*29*	*20.1*	*21*	*13.1*	*0.121*	*7*	*25.9*	*22*	*18.8*	*0.429*
	Absent	*115*	*79.9*	*139*	*86.9*		*20*	*74.1*	*95*	*81.2*	
Ways of antibiotic usage	Completely Used	*20*	*69.0*	*13*	*65.0*	1.00	*5*	*71.4*	*15*	*68.2*	1.00
	Not-Completely Used	*9*	*31.0*	*7*	*35.0*		*2*	*28.6*	*7*	*31.8*	
Present presence of respiratory infection	Present	*15*	*10.4*	*16*	*10.0*	1.00	*5*	*18.5*	*10*	*8.5*	0.159
	Absent	*129*	*89.6*	*144*	*90.0*		*22*	*81.5*	*107*	*91.5*	
Number of family members	2-4	*46*	*31.9*	*55*	*34.4*	0.868	*9*	*33.3*	*37*	*31.6*	1.00
	5-6	*70*	*48.6*	*73*	*45.6*		*13*	*48.1*	*57*	*48.7*	
	>6	*28*	*19.4*	*32*	*20.0*		*5*	*18.5*	*23*	*19.7*	
Presence of health institution worker in the family	Present	*17*	*11.8*	*10*	*6.3*	0.107	*2*	*7.4*	*15*	*12.8*	0.531
	Absent	*127*	*88.2*	*150*	*93.8*		*25*	*92.6*	*102*	*87.2*	
Family member hospitalization in the past 1 years	Present	*18*	*12.5*	*14*	*8.8*	0.289	*8*	*29.6*	*10*	*8.5*	0.007
	Absent	*126*	*87.5*	*146*	*91.3*		*19*	*70.4*	*107*	*91.5*	
Number of children in class room	21-40	*29*	*20.1*	*57*	*35.6*	*0.000*	*5*	*18.5*	*24*	*20.5*	0.016
	41-60	*70*	*48.6*	*64*	*40.0*		*8*	*29.6*	*62*	*53.0*	
	>60	*45*	*31.3*	*39*	*24.4*		*14*	*51.9*	*31*	*26.5*	

Where: *CONS* Coagulase-negative *Staphylococcus, MRSA* Methicillin Resistance *S.aureus.*

Of the total 27 MRSA isolates, the maximum isolates (59.9%, 1 4) were from those children that belongs to class rooms containing greater than 60 children, while (29.6%, 8), and (18.5%, 5) of the MRSA were isolated from those children selected from class rooms with class size of 41–60 and 21–40 children, respectively. *S. aureus* (p=0.000) and MRSA (p=0.016) nasal carriages were significantly associated to the number of children in the class room. Similarly, MRSA nasal carriage were the highest among children that came from households with 5–6 family members (48.1%, 13), followed by 2–4 family members (33.3%, 9) and >6 family members (18.5%, 5). However, there was no significant relationship between number of members of the house hold to S*. aureus* (p=0.868) and MRSA (p=1.00) nasal carriage.

Majority of the carriers for nasal *S. aureus* (79%, 115) and MRSA (74.1%, 20) among the primary school children were those who did not use antibiotics during the last 4 weeks before sample collection. Neither *S. aureus* (P=0.121) nor MRSA (p=0.429) were significantly associated to child’s antibiotic usage.

The rates of isolation of MRSA from children with respiratory infection were (18.5%, 7). Majority of *S. aureus* (89.6%, 129) and MRSA (81.5%, 22) carriers’ did not have respiratory infection during sample collection. Presence of respiratory infection was not significantly related to *S. aureus* (p=1.0) and MRSA (p= 0.159) nasal carriage among the children. From among 27 children who carried MRSA, only (7.4%, 2) of the children family had relation with health institution while (92.6%, 25) did not. *S. aureus* (p=0.107) and MRSA (p= 0.531) nasal carriages were not significantly associated with presence or absence of health institution worker in the child’s family (Table 6).

Examination of risk factors for prevalence of MRSA among the prisoners has revealed three major factors: the first was high number of prisoners in dormitory (p=0.034) (Table 7). All MRSA carriers 12(100%) were isolated from those prisoners that live with greater than 60 jail inmates within single room. The 2^nd^ risk factor associated with MRSA nasal carriage was having antibiotic therapy 4 weeks prior to positive culture results (p=0.030*).* Of the 12 MRSA isolates, 6(50%) were isolated from those prisoners that had history of antibiotic therapy 4 weeks prior to positive cultures. The 3^rd^ risk factor was presence of respiratory infection during sample collection (p=0.005*)*. Accordingly, of the total 12 MRSA isolates, 7(58.3%) were from those prisoners who had respiratory infection. All other risk factors did not show statistical significance for MRSA nasal carriage (Table 7). As observed in this investigation, age (p=0.005*)* and high number of jail inmates in a dormitory (p=0.034*)* were the two statistically significant risk factors for high rate of *S. aureus* nasal carriage among the prisoners. *S. aureus* and MRSA carriage were higher among the age group of 18–25 (Table 7). All other risk factors did not show statistical significance for *S. aureus* nasal carriage.

**Table 7:** **Analysis of epidemiologic risk factors for *S. aureus *and MRSA nasal carriage among prisoners, Jimma town, 2011**

**Risk factors**			***Staphylococcus *N=50**		**P**	**Methicilin-resistant *S. aureus *n=25**		**p**
			***S.aureus***	**CNS**		**Positive**	**Negative**	
Age	18-25	No.	20	9	0.005	9	11	0.787
		%	80.0	36.0		75.0	84.6	
	26-35	No.	2	9		1	1	
		%	8.0	36.0		8.3	7.7	
	36-45	No.	3	7		2	1	
		%	12.0	28.0		16.7	7.7	
Sex	Male	No.	25	25	-	12	13	-
		%	100	100		100	100	
Previous hospitalization in the past 1 year	Present	No.	5	2	0.417	2	3	1.00
		%	20	8		16.7	23.1	
	Absent	No.	20	23		10	10	
		%	80.	92		83.3	76.9	
Antibiotic usage in the past 4 weeks	Present	No.	7	6	0.5	6	1	0.030
		%	28	24		50	7.7	
	Absent	No.	18	19		6	12	
		%	72	76		50	92.3	
Extent of usage of subscribed antibiotics	Completed	No.	5	6	0.209	4	1	1.00
		%	62.5	100		66.7	50	
	Not-Completed	No.	3	-		2	1	
		%	37.5	-		33.3	50	
Having respiratory infection at time of study	Present	No.	11	13	0.778	9	2	0.005
		%	44	52		75	15.4	
	Absent	No.	14	12		3	11	
		%	56	48		25	84.6	
Number of prisoners in dormitory/room	1-20	No.	1	3	0.048	-	1	0.034
		%	4	12		-	7.7	
	21-40	No.	1	6		-	1	
		%	4	24		-	7.7	
	41-60	No.	4	6		-	4	
		%	16	24		-	30.8	
	>60	No.	19	10		12	7	
		%	76	40		100	53.8	
Presence of hospital admitted patients in prisoner room(in the last 1yr)	Present	No.	8	12	0.387	5	3	0.411
		%	32	48		41.7	23.1	
	Absent	No.	17	13		7	10	
		%	68	52		58.3	76.9	

N= total number of nasal swabs taken from prisoners, n= total number of Methicillin-Resistant *S. aureus* isolates from prisoners.

## Discussion

Approximately 20-30% of healthy persons are persistent carriers of *S. aureus* and 60% are intermittent carriers with high colonization rates among risk groups including hospital patients, children’s and jail inmates [13,14]. In the present study, a total of 354 nasal swabs were collected from 304 primary school children and 50 prisoners. The frequency of isolation of *S. aureus* from primary school children of Jimma town was 47.3%, with an overall frequency of isolation being 47.74% (169/354). In a related study, Yildirim *et al*. [15] also reported an isolation rate of 37% from nasal swabs of primary school children aged 6–14 years. According to Rijal, *et al*. [10], the rate of isolation of *S. aureus* from school children was 31%. The relatively low isolation rate reported in the later two studies could be due to the direct inoculation of the nasal swabs on Mannitol salt agar without primary and secondary enrichment which may reduce the possible isolation rate of the organism. According to the present study, the isolation rate of *S. aureus* from among prisoner was 50%. Worldwide isolation rate of *S. aureus* from among prisoners was reported to range from 40.5% to 78% [13,14].

In the present study, the prevalence of MRSA among primary school children aged 5–15 years was 18.8%. Here, MRSA was detected using Cefoxitin (30μg) disc which has high efficiency to detect MRSA and has been used as an alternative to PCR in resource constrain areas [12,16]. Alfaro *et al*. [17] also reported 22% carriage rate of MRSA among children of south Texas. The findings of this study was in agreement with earlier studies reported from different parts of the world [10,12].

The prevalence of MRSA among prisoners of Jimma town was 48%, the first report of its kind from Ethiopia. A recent report from Philippine showed 41% prevalence of MRSA among inmates of Manila city jail [14]. In another study conducted at California, the prevalence of MRSA was reported to be 54% [18]. In the present study, high nasal colonization of *S. aureus* and MRSA among primary school children and prisoners could be accounted to the associated risk factors. The main risk factors for primary school children of the study area were age, sex, number of children per class room, child’s and family member’s previous year hospitalization. As clearly shown in the result section, *S. aureus* (p=0.000) and MRSA (p=0.016) nasal carriage was significantly associated to the number of children per class room with the highest prevalence of *S. aureus* (30.6%, p=0.011) and MRSA (48%, p=0.027) being in class rooms where the number of children was greater than 60. Furthermore, MRSA nasal carriage was the highest among children that came from households with 5–6 family members. A similar study indicated that *S. aureus* and MRSA nasal carriage is the highest (62.06%) among students belonging to age group of 6–10 [10].

The current study shown that MRSA nasal carriage was the highest among female children compared to male children (23[85.2%] Vs 4 [14.8%]; p=0·000). Similarly, Rijal, *et al*. [10] reported higher prevalence of MRSA in female children than males (22[68.7%] Vs 10[31.3%]). In the study conducted at Primary School in Düzce, Turkey, Yildirim, *et al*. [15] also reported higher prevalence of MRSA among female children when compared to male counterpart.

In this study, 29.6% of MRSA strains were isolated from children that had history of previous year hospitalization (p=0.007) and also 14.8% from children that had family member hospitalized in previous year (p=0.041). Many internationally accepted definitions of hospital acquired MRSA (HA-MRSA) emphasize the isolation of MRSA from an individual that has history of previous year hospitalization [19-22]. Therefore, isolation of MRSA from an individual that had risk factor of HA-MRSA in the present study and other similar reports of high prevalence of MRSA from outpatient and healthy staff member [9,23] could indicate the dissemination of HA-MRSA from hospital to the community outside hospital. Such condition contributes to high prevalence of MRSA outside hospitals.

As compared to the school children, Epidemiologic risk factors that have significant association with MRSA nasal carriage among prisoners were: high number of prisoners (>60) per dormitory/room (p=0.0340), antibiotic usage (p= 0.01) and presence of respiratory infection (p=0.005). Similar studies showed that the rate of nasal colonization of MRSA is high when there are predisposing risk factors like antibiotic usage in previous 4 weeks and presence of respiratory infection during sample collection which was supposed to be risk factors for HA-MRSA [13,24].

All *S. aureus* isolates showed 100% resistance to Penicillin. Similarly, MRSA isolates showed 100% resistance to Ampicillin, Penicillin and Cefoxitin. In agreement with this observation, Gabriel and Kebede also reported 100% resistance of *S. aureus* isolated from among Health Workers of Jimma University Specialized Hospital [9]. Likewise, Uwaezuoke and Aririatu [25] reported high resistance to Penicillin (95.8%) of *S. aureus* strains isolated from clinical sources in Owerri, Nigeria. The absolute resistance of MRSA isolates to these antibiotics indicates the dissemination and dominance of HA-MRSA in the community, hence CA-MRSA strains, capable of resisting only β- lactam antibiotics as the result of carriage of genetic element SCCmec typeIV. *SCCmec* type IV is one of the shorter SCC*mec* variations less likely to carry multi-drug resistance [26]. Significant proportions of MRSA isolates (87.2%) were susceptible to Vancomycin (p=0.000), Gentamycin (84.6%) (p=0.006) and Trimethoprim-sulfamethoxazole (82.1%) (p= 0.000). Among *S. aureus* isolates, 97% were susceptible to Vancomycin, 95.3% to Gentamycin and 94.7% to Trimethoprim-sulfamethoxazole. In review of similar work, Uwaezuoke and Aririatu [27] reported the degree of susceptibility of *S. aureus* to Vancomycin and Gentamycin to be as high as 91.7%. Similarly, Rijal, *et al*. [10] reported 96.9% susceptibility of *S. aureus* isolates to Vancomycin.

The intensive use of the antibiotics in the study area could account for the possible isolation of hospital type MRSA in the community that have developed resistance to many types of antibiotics and also indicates the transfer of these resistant genes to the microbial community outside the hospital. The resistances of MRSA to non β-lactam antibiotics were the characteristics of HA-MRSA [28]. With this regards, our result shows the mixing of HA-MRSA and CA-MRSA in the community. Several researchers [27,29] identified the possible mixing of HA-MRSA and CA-MRSA. Likewise, Klevens *et al*. [29] also reported the mixing of CA-MRSA with HA-MRSA in intensive care unit patients after studying MRSA rates and trends between 1992 and 2003, the result that showed the rise in HA-MRSA from 35.9% to 64.4%. The same authors also showed a significant decrease in MRSA resistance to non-β-lactam antibiotics including Gentamycin, Tetracycline and Trimethoprim-sulfamethoxazole and significant increase in MRSA infection. This indicates that HA-MRSA is also responsible for increase in prevalence of CA-MRSA because of significantly related risk factors. The risk factors investigated in our present work are responsible for high rate of transmission of HA-MRSA from Hospital to the community in Jimma town.

In general, the present study revealed high CA-MRSA both among primary school children and inmates in jail of Jimma zone, calling for appropriate surveillance for drug resistance patterns of microbial isolates for commonly used antibiotics not only within the health institutions, but also the community at large.

## Competing interests

The authors have no competing interests to declare.

## Authors’ contributions

TK involved in designing of the project, collection of data, data analysis and interpretation, and write up of the manuscript. KB designed the study, supervised data collection both on field and in laboratory, and prepared the manuscript for publication. Both authors read and approved the final manuscript.
